# Effectiveness of a Multicomponent Group Psychological Intervention Program in Patients with Inflammatory Bowel Disease: A Randomized Trial

**DOI:** 10.3390/ijerph18105439

**Published:** 2021-05-19

**Authors:** Purificación Bernabeu, Carlos van-der Hofstadt, Jesús Rodríguez-Marín, Ana Gutierrez, Miguel Raúl Alonso, Pedro Zapater, Rodrigo Jover, Laura Sempere

**Affiliations:** 1 Health Psychology Department, Hospital General Universitario de Alicante, Instituto de Investigación Sanitaria ISABIAL, 03010 Alicante, Spain; cjvander@umh.es (C.v.-d.H.); mralonsog@gmail.com (M.R.A.); 2 Departamento de Psicología de la Salud, Universidad Miguel Hernández, 03202 Elche, Spain; rod.marin@umh.es; 3 Servicio de Medicina Digestiva, Hospital General Universitario de Alicante, Instituto de Investigación Sanitaria ISABIAL, 03010 Alicante, Spain; gutierrez_anacas@gva.es (A.G.); rodrigojover@gmail.com (R.J.); lausemro@hotmail.com (L.S.); 4 Clinical Pharmachology Department, Hospital General Universitario de Alicante, Instituto de Investigación Sanitaria ISABIAL, 03010 Alicante, Spain; zapater_ped@gva.es

**Keywords:** inflammatory bowel disease, psychological intervention, disease activity

## Abstract

(1) Background: Stress, anxiety, and depression have been identified as factors that influence the development of inflammatory bowel disease (IBD). The main aim of this study was to test the effectiveness of group multicomponent cognitive-behavioral therapy at reducing stress, anxiety, and depression, and improving quality of life and the clinical course of the disease. (2) Methods: A total of 120 patients were evaluated using the General Perceived Stress Scale, Scale of Stress Perceived by the Disease, the anxiety and depression scale, and quality of life questionnaire for patients with IBD. Disease activity was measured using the Mayo Index for ulcerative colitis and CDAI for Crohn’s disease, as well as the number of relapses self-reported by patients. Patients were randomized to receive group multicomponent cognitive-behavioral therapy or treatment as usual. (3) Results: The psychological intervention reduced stress (EAE: 45.7 ± 8.8 vs. 40.6 ± 8.4, *p* = 0.0001; PSS: 28.0 ± 7.3 vs. 25.1 ± 5.9, *p* = 0.001) and improved quality of life (164.2 ± 34.3 vs. 176.2 ± 28.0, *p* = 0.001). An improvement was found in the number of relapses self-reported by patients (0.2 relapses/patient vs. control 0.7 relapses/patient; *p* = 0.027). No differences were found in disease activity indexes. (4) Conclusions: Psychological therapy was associated with improved stress, quality of life and with a decrease in the number of relapses self-reported by patients. Clinical trial registration number: NCT02614014.

## 1. Introduction

Different studies have shown the influence of psychological and social factors on the course, clinical expression, and relapse of inflammatory bowel disease (IBD) [1,2]. On the one hand, some studies have concluded that patients with IBD have a higher rate of affective alterations than the general population, especially regarding anxiety and depression [3,4,5,6]. However, other recent psycho-immunological studies have demonstrated the role of stress and emotional disorders in the pathobiology of IBD [7].

Several studies have evaluated the role of psychological intervention in the course of IBD [8,9,10,11]. However, these studies were highly heterogeneous in terms of psychological techniques and intervention models, and sometimes biased by methodological limitations, making it difficult to draw clear conclusions. In general, interventions based only on stress management have demonstrated a modest benefit in mental symptoms or quality of life [12]. Cognitive-behavioral therapies have provided the greatest benefit to patients with IBD in terms of reducing symptoms of anxiety and depression and improving patients’ quality of life, but only a small benefit in reducing IBD symptoms [8]. Wietersheim and Kessle [13] concluded that, though psychological intervention can improve psychological symptoms, it has no impact on the course of the disease. Recent studies with mindfulness therapy have also shown efficacy in reducing anxiety symptoms and improving quality of life in patients with IBD [14].

We thought it was interesting to use a clinical trial as the study design, a model that is not very common in psychology. This model has its origins in psychology in Wundt’s experimental model, from which conventional psychology began to be considered a natural science. 

The aim of the present study was to test the effectiveness of group multicomponent cognitive-behavioral therapy (MCBT) in patients with IBD in a randomized controlled trial (RCT) in an attempt to overcome the possible bias found in previous studies. MBCT is focused on the association between thought and behavior. The therapy usually combines cognitive restructuring techniques, relaxation training, and other coping strategies. In our study, we combined this therapy with third-generation psychological techniques, such as mindfulness or the Barlow protocol for anxiety and depression. We worked on the basis that cognitive, affective, and behavioral aspects are interrelated; thus, a change in one of them affects the other two components.

Our primary goal was to determine the effect of MCBT on stress, anxiety, depression, and quality of life. The secondary goal was to test whether this MCBT is able to improve the activity of the disease measured by both: the activity index and the number of flares of the disease. 

## 2. Materials and Methods

We performed a randomized controlled trial registered at clinicaltrials.gov under the number NCT02614014. The study was conducted in the Inflammatory Bowel Disease Unit at Hospital General Universitario de Alicante between January 2016 and December 2018. The duration of the study was 3 years to elaborate the program (January–June 2016), complete patient recruitment and implementation of the program (June 2016–December 2017), and take post-intervention measurements (until December 2018). All the authors had access to the study data and reviewed and approved the final manuscript.

### 2.1. Study Population

A total of 120 patients with a diagnosis of IBD were enrolled in parallel and allocated 1:1. Inclusion criteria were a diagnosis of Crohn’s disease (CD) or ulcerative colitis (UC), age > 18 years, a minimum score of 300 on the Stressful Life Events Inventory (SRRS) as a measure of propensity to stress, and autonomous in completing assessment questionnaires and treatment materials. Exclusion criteria were clinically inactive IBD in the last 18 months, severe psychiatric disorder or current psychotherapy.

After medically screening the patients, they were administered a semi-structured interview to provide information about gender, age, education, marital and employment status, medical treatments, and history of relapse. A test battery was then administrated to measure the Perceived Stress Scale (PSS) [15], perceived stress from the illness (EAE) [16], life events inventory (SRRS) [17], hospital anxiety and depression scale (HADS) [18], and quality of life (IBDQ) [19].

To measure CD activity, we used the Clinical Disease Activity Index (CDAI) [20], and to measure UC activity, we used the partial Mayo Score [21]. Clinical disease activity was also measured through relapses reported by patients. For this purpose, we considered any increase in disease activity as perceived as such by the patient (changes in frequency of defecation, pain, bleeding, etc.) that would prevent him/her from doing their activities normally, with or without hospital admission, as a flare in disease activity. Patients were instructed to self-register these increases in disease activity in a patient diary.

**Figure 1:**
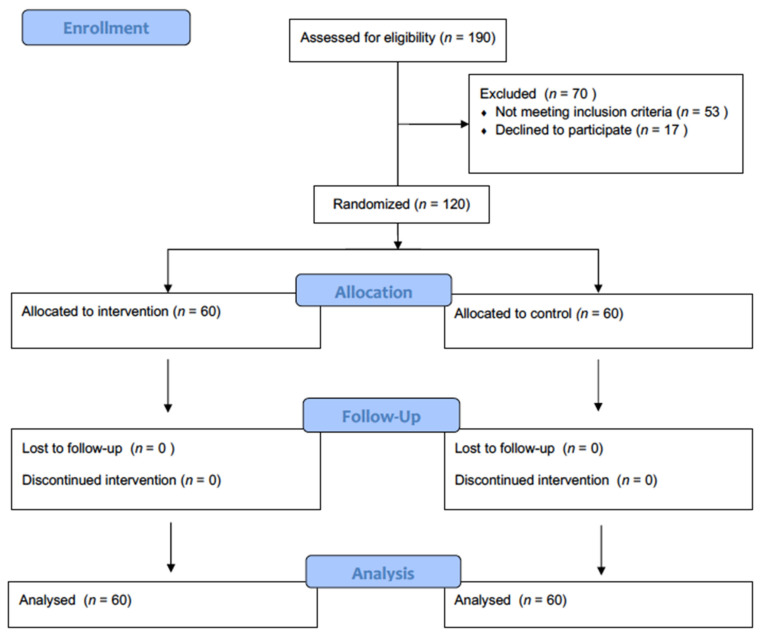
Flow-chart of the study.

Patients were randomized to psychological intervention plus usual care or only usual care (Figure 1). Sixty patients were assigned to the control group (treatment as usual) and 60 to the experimental group (multicomponent group psychological intervention program plus usual care). The psychological intervention was always conducted by trained psychologists. Randomization was performed using the website https://numero-aleatorio.com/generadores/ (accessed on 5 September 2016). 

### 2.2. Outcomes

Primary outcomes were improvement in measures of stress (PSS and EAE scales), anxiety and depression (HAD scale) and quality of life (IBDQ scale).

Secondary outcomes were improvement in measures of disease activity (CDAI and Mayo score) as well as the number of disease relapses during follow-up.

### 2.3. Ethical Considerations

The study was conducted in accordance with Spanish Law 14/2007 of 3 July on Biomedical Research, the Helsinki Declaration of the World Medical Association (1964, revised in 2013), the standards of Good Clinical Practice, and the legislation in force in this area. All patients included in the study read the patient information sheet and signed the informed consent form. All patient data were treated anonymously by assigning a double code to both the sample and the data file, and only duly authorized personnel had access to personally identifiable data. The present study was submitted for evaluation and approved by the IRB of Hospital General Universitario de Alicante.

### 2.4. Psychological Intervention: Characteristics of the MCBT Program

**Figure 2:**
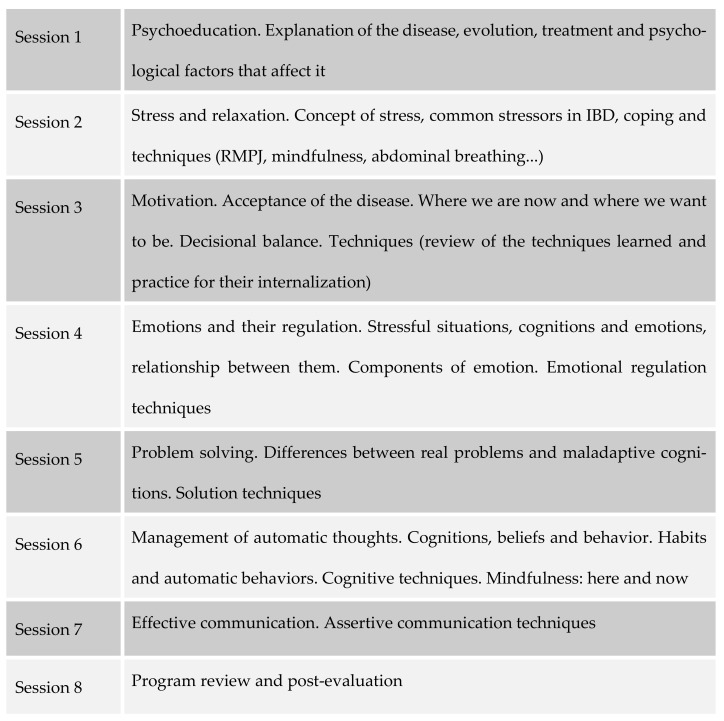
MCBT Program.

Patients were randomized to psychological interventions consisting of eight sessions, once per week, of 90-minute duration. Each group had a total of 8 to 10 patients. The psychological intervention program was self-made following the guidelines of the cognitive-behavioral model (Figure 2).

The sessions were structured following this schedule: review previous session’s tasks, check progress, main theme of the session, new tasks and doubts. The program followed the guidelines of the cognitive-behavioral model, extending the contents with third-generation techniques, such as mindfulness and emotional regulation, according to the transdiagnostic model of Barlow [22]. The aim of this program is to bring together all of the techniques that demonstrated effectiveness in certain areas and use them together to cover a more global intervention. Following these guidelines, the first session was psychoeducation, in which the patient was given an explanation of everything related to their illness that could alleviate their uncertainty and initial doubts and show that they could generate fear and anxiety, causing, in some cases, symptomatic hypervigilance, erroneous cognitive anticipations, and demand for medical assistance, which was sometimes unnecessary. The second session was about the dimensional model of pain and how stress, anxiety, and depression can negatively affect our perception of illness/health. The concept of stress was also discussed in all its dimensions: cognitive, physiological, emotional, and behavioral. Potential stressors affecting chronic diseases were exposed and we helped patients identify their own stressors—both life events and everyday stress. We then evaluated adaptive coping strategies and began to use relaxation techniques—both muscle relaxation and mindfulness. The third session dealt with the issue of empowerment; we tried to involve the patient as an active agent to achieve their own well-being and improve their quality of life. In the fourth session, we dealt with emotional regulation, following Barlow’s model from a common transdiagnostic perspective for different emotional disorders. In the fifth session, we identified problems that may be affecting our health, sometimes by generating anticipatory anxiety. In the sixth session, we instructed patients to identify his or her own maladaptive thoughts and how these often appear uncontrolled. Mental control was worked through mindfulness techniques. In the seventh session, we continued with mindfulness as an instrument to reduce reactivity to stress, anxiety, and depression symptoms. In the eighth session, all of the techniques were reviewed and the benefit they can obtain through continued practice was emphasized. At the end of the session, a post-intervention evaluation was carried out with all of the tests administered in the pre-test. The disease activity was also determined again by calculating the activity indexes, as well as the number of relapses of activity reported by the patient during the period between the initial evaluation and the end of the intervention process. At the same time, patients assigned to the control group were called for follow-up evaluation.

### 2.5. Statistical Analysis

Since psychological intervention is primarily aimed at reducing stress levels, the effect on stress has been used to calculate the sample size, assuming that this reduction in stress will translate into an improvement in the clinical activity of IBD. To calculate the sample size, we assumed a reduction in the average baseline score of 10% for stress in the EAE measurement in the intervention group and a coefficient of variation of 0.25 with a power of 80%. Using a paired Student’s t test with a significance level of 0.05, we would need to include 57 patients per group.

Continuous variables were expressed as mean ± standard deviation (SD) and categorical variables as frequency and percentage. The assumption of normality was established through the Kolmogorov–Smirnov test. Differences between the baseline characteristics of the control and intervention groups were analyzed using the paired Student’s t test for independent measures in the case of quantitative variables and McNemar’s chi-squared test to measure the association between qualitative variables. Differences between baseline and post-intervention outcomes were analyzed using the Student’s t test for paired samples. We also used the General Linear Model for comparison between baseline and post-intervention outcomes. For multiple comparisons, the significance value was adjusted using the Bonferroni correction. Non-normally distributed variables were compared using the Mann–Whitney U test. Hypothesis contrasts were raised with a significance level of 5% (*p* = 0.05) in the statistical package SPSS V22.0 (IBM Corp. Relayed, 2013). Effect size was estimated for quantitative variables, with the Cohen d statistic interpreted with Cohen criteria for all significant differences and qualitative variables with Cramér’s V coefficient.

## 3. Results

### 3.1. Baseline Characteristics

The baseline characteristics of patients are given in Table 1. The sample was homogeneous, with no significant differences between the intervention and control groups. In addition, no differences were found in psychometric or quality of life variables at baseline.

### 3.2. Effectiveness of the Program

The median number of days between baseline assessment and post-intervention assessment was 237 days (25th-75th interquartile range 155–358 days). We found no difference in this variable between the control and intervention groups (median 266 days vs. 190 days, respectively; *p* = 0.06).

Analysis of the data obtained from the comparison between pre- and post-intervention measures (Table 2) found that patients in the intervention group improved significantly in terms of the general measures of stress EAE and PSS. However, this improvement was not observed in the control group (EAE control: pre 45.4; post 44.4; *p* = 0.352; intervention: pre 45.7; post 40.6; *p* = 0.0001. PSS control: pre 26.7; post 26.0; *p* = 0.466; intervention: pre 28.0; post 25.1; *p* = 0.001). Using the General Linear Model, we also found significant differences in post-intervention outcomes in EAE and PSS. However, these differences did not reach statistical significance for the interaction between control–intervention and pre–post intervention for PSS (Table 2). However, both groups improved significantly in anxiety and depression scores (Table 2).

Regarding perceived quality of life, significant improvement was observed in the intervention group versus control group (IBDQ control: pre 164.9; post 170.6; *p* = 0.149; intervention: pre 164.2; post 176.2; *p* = 0.001). This improvement is significant in the overall quality of life score, particularly in the social and emotional post-treatment dimensions in the intervention group. Using the General Lineal Model, we also found significant differences in pre–post intervention in IBDQ total, IBDQ social and IBDQ emotional, but these differences were not statistically significant for the interaction between control–intervention and pre–post intervention (Table 2).

### 3.3. Effect of the MCBT Program on Disease Activity

Disease activity was measured in two different ways: by the disease activity index (CDAI and partial Mayo Score, Table 3) and by the number of relapses reported by patients before and after the intervention (Table 4). We found no differences regarding activity index in CD, with improvement in both the control (139.7 ± 98.7 vs. 97.3 ± 72.9, *p* = 0.031) and intervention group (157.6 ± 125.5 vs. 131.0 ± 111.2, *p* = 0.004). No improvement was seen in UC patients (control 2.2 ± 2.2 vs. 1.3 ± 2.3, *p* = 0.190; intervention 1.8 ± 2.1 vs. 1.5 ± 2.4, *p* = 0.589) (Table 3). However, when we measured the activity based on the number of disease flares reported by patient, we found that the number of relapses per patient (control 0.7 vs. intervention 0.3; *p*= 0.027) and relapses per month perceived after treatment (control 0.07 vs. intervention 0.03; *p* = 0.034) was significantly reduced in the intervention group compared to the control group (Table 4). In addition, the proportion of patients with disease relapse was higher in the control group (control 30% vs. intervention 10%; *p* = 0.036).

## 4. Discussion

The main result of our study is that group MCBT performed in patients with IBD was able to significantly reduce the levels of stress attributable to the disease; this was the primary outcome of the study. Our results also show an improvement in the quality of life of these patients compared to controls with no intervention and treatment as usual. However, regarding the secondary outcome of our study, our results are not entirely conclusive, as we have not been able to demonstrate differences in improvement in the disease activity index, though there was an improvement in the number of relapses self-reported by the patients. The main strengths of our study are the RCT design and selection of patients with recent disease activity and a moderate-high baseline level of stress.

Psychological distress is frequent in patients with IBD [11,23,24]. Recent studies with longitudinal data from more than 2000 subjects have found that symptoms of depression and anxiety may have a strong correlation with clinical recurrence of IBD [25]. These variables usually coexist with psychological stress, which has also been widely investigated in the context of IBD. The role and impact of recent stressful life events have been documented as important factors associated with psychopathological symptoms in patients with IBD [26]. Other authors have reported a significant association between symptoms of depression, anxiety, or stress and clinical relapse of IBD [25]. There is agreement that patients with IBD should be screened for clinically significant distress and referred to mental health practitioners for further evaluation and treatment [27].

However, the effect of psychological intervention on the evolution of chronic diseases is controversial. Specifically in IBD, there are conflicting results. A Cochrane review [28] concluded that, in general terms, psychological intervention has no effect on patients’ quality of life, emotional status, or the proportion of patients in remission. However, this review recognizes profound limitations due to the high heterogeneity of the included studies, the high risk of methodological biases, and the interpretation of the results. Therefore, new studies focused on specific therapeutic aspects and specific patient groups are recommended. Given this high heterogeneity, Knowles et al. [12] recommended some requirements that studies on psychological interventions in IBD should meet in order to avoid methodological biases. In the present study, we have met these requirements: The design has been controlled and randomized; a power analysis was performed to calculate the sample size; the study is replicable using the same instruments, which are available to other researchers; and both the evaluation and the intervention were carried out by appropriate and qualified professionals. Therefore, in this study, we overcome the biases of some studies in which the program was taught by professionals from disciplines other than psychology or psychiatry [8,29,30]. Our results show that psychological intervention with MCBT, which includes cognitive restructuring, relaxation, mindfulness and coping skills, improves stress in IBD. This particular psychological therapy can be optimal for these patients given the particular characteristics of psychological impairment found in IBD, with predominance of stress as a trigger of disease activity.

Regarding quality of life, the results of our study indicate a significant improvement in IBDQ score after the intervention; however, our sample size did not have enough power to detect these differences in the more stringent general linear model. These results reproduce those obtained in other studies [31,32]. Along the same lines, Hunt et al. [15] reported an improvement in health-related quality of life in a group of patients attending self-help cognitive-behavioral therapy. A recent review and meta-analysis [10] found that psychological therapy, and cognitive-behavioral therapies in particular, had beneficial effects on depression and quality of life in patients with IBD, but no impact on objective disease activity.

Previous studies [28,30,32] indicate that it is difficult to demonstrate the effect of psychological intervention on a hard variable, such as disease activity, which is influenced by multiple causes. Our results are in line with others, which were not able to demonstrate an effect on the disease activity index. However, we saw a clear influence of psychological intervention in a subjective measure of activity, such as the patient self-reported number of relapses. This form of data collection may suffer some limitations when evaluating the measured variable and, clearly, is not a perfect source of information. However, this is a patient-reported outcome measure (PROM) that can have high value from the patient’s point of view. In addition, the Food and Drug Administration and other organizations are moving from the CDAI to PROMs and objective measures of disease, such as findings from endoscopy [33]. In that case, taking into account the self-reported disease activity, our psychological program is able to reduce the disease activity perceived by the patient. On the other hand, the lack of effect found in the activity index could be explained by the fact that disease activity is influenced by multiple factors such as medication, diet, infections or smoking, stress being only one of these factors to be taken into account.

## 5. Conclusions

In summary, the results of the present study have demonstrated the effect of MCBT on reducing stress and improving quality of life. Our results can only be applied to patients with propensity to stress, and the results of our study should be restricted to this class of patients. Moreover, this program has an effect on patient self-reported disease activity. Our study has been designed to overcome most of the biases that prevent concluding that psychological treatment can effectively benefit these patients. As IBD is a disease of unknown origin, but stress, anxiety, and depression may be risk factors, it would be beneficial to implement group psychological treatment in the protocol of care for these patients.

## 6. Limitations

A limitation of this study is the lack of long-term follow-up of our patients; however, this follow-up is being conducted now and will be included in future reports. Moreover, we have not used objective markers for disease activity, such as CRP, fecal calprotectin levels or endoscopy, because these kinds of tests might make enrolment significantly more complex. However, especially in CD, we know that clinical symptoms could very poorly correlate with disease activity. Moreover, variation in the assessment of outcomes can introduce some changes in the measurement of effects. Finally, we included patients with different degrees of disease activity. Although the influence of psychological intervention on disease activity was an outcome of the study, the design aimed to obtain a clearer picture of the role of psychological intervention in the course of the disease. Moreover, it would be interesting to include and treat patients with confirmed active disease and test the effect of MCBT; this has been an unrealistic way to try to evaluate the role of psychological intervention in patients with a flare of disease, because the development of disease activity would likely be influenced more by medical treatment, and a more heterogeneous sample of patients would be included.

## Figures and Tables

**Table 1:** Baseline measurements and demographic characteristics.

Socio-Demographic Variables	Intervention (*n* = 60)	Control (*n* = 60)
Age (mean ± SD)	44.5 ± 11.81	42 ±11.65
Gender, *n* (%)		
Male	28 (46.7%)	19 (31.7%)
Female	32 (55.6%)	41 (68.3%)
Marital status, *n* (%)		
Married/cohabited	37 (61.7%)	45 (75.0%)
Single	14 (23.3%)	9 (15.0%)
Widower	1 (15.0%)	2 (3.3%)
Divorced	0	4 (6.7%)
Academic level, *n* (%)		
Unschooled	2 (3.3%)	1 (1.7%)
Primary	13 (21.7%)	15 (25.0%)
Secondary	25 (41.7%)	24 (40.0%)
Superiors	20 (33.3%)	20 (33.0%)
Employment situation, *n* (%)		
Working		
	31 (51.7%)	22 (36.7%)
Unemployed		
	14 (23.3%)	22 (36.7%)
Retired		
	12 (20%)	11 (18.3%)
Housewife		
	3 (5.0%)	5 (8.3%)
Diagnosis, *n* (%)		
Crohn’s disease	43 (71.7%)	37 (61.7%)
Ulcerative colitis	17 (28.3%)	23 (38.3%)
Bowel resection, *n* (%)	16 (26.7%)	11 (18.9%)
Age at diagnosis (mean ± SD)	34.3 ± 12.6	31.5 ± 11.9
Time since diagnosis (mean ± SD)	9.9 ± 8.5	10.7 ± 8.8
Active Smokers *n* (%)	12 (20.0%)	20 (33.3%)
No. of flares (mean ± SD)	19.8 ± 27.3	26.5 ± 47.1
CDAI (mean ± SD)	157.6 ± 125.5	139.7 ± 98.7
Non-active disease, *n* (%)	24 (56.8%)	21 (56.8%)
Active disease, *n* (%)	19 (44.1%)	16 (43.2%)
Mayo score (mean ± SD)	1.8 ± 2.1	2.2 ± 2.2
Non-active disease, *n* (%)	16 (94.1%)	19 (82.6%)
Active disease, *n* (%)	1 (5.9%)	4 (17.4%)
*Psychological variables*		
		
EAE total, mean ± SD	45.7 ± 8.9	45.4 ± 9.4
PSS (mean ± SD)	28.0 ± 7.3	26.7 ± 7.7
SRRS (mean ± SD)	317 ± 151.1	315 ± 123.06
HAD		
Anxiety (mean ± SD)	8.1 ± 4.1	8.4 ± 4.8
Depression (mean ± SD)	5.1 ± 3.5	5.5 ± 4.5
IBDQ		
IBDQ total (mean ± SD)	164.2 ± 34.3	164.9 ± 37.6
		
IBDQ digestive (mean ± SD)	54.5 ± 10.3	53.2 ± 12.8
IBDQ systemic (mean ± SD)	22.8 ± 5.9	22.7 ± 6.6
IBDQ social (mean ± SD)	27.1 ± 7.9	28.9 ± 6.7
IBDQ emotional (mean ± SD)	59.7 ± 13.4	60.1 ± 15.2

EAE: Disease-related Stress Scale; HAD: Hospital anxiety and depression scale; PSS: Perceived Stress Scale; SRRS: Scale of Stressful Life Events; IBDQ: quality of life in IBD. SD: Standard Deviation.

**Table 2:** Effectiveness of the group psychological intervention program on psychological variables and quality of life.

Psychological Variables	Group	Pre-Intervention	Post-Intervention	*p*	ES (gl = 59)
EAE	Control	45.4 ± 9.4	44.4 ± 9.7	0.352 0.000 ^a,b,c,d^	0.58 ^e^
	Intervention	45.7 ± 8.8	40.6 ± 8.4		
PSS	Control	26.7 ± 7.7	26.0 ± 9.9	0.466	-
	Intervention	28.0 ± 7.3	25.1 ± 5.9	0.001 ^a,c^	0.41 ^e^
Anxiety	Control	8.4 ± 4.8	7.2 ± 5.0	0.009	0.33 ^e^
	Intervention	8.1 ± 4.1	6.5 ± 3.9	0.003 ^a,c^	0.38 ^e^
Depression	Control	5.5 ± 4.5	4.4 ± 4.2	0.012 ^a^	0.32 ^e^
	Intervention	5.1 ± 3.5	4.1 ± 3.4	0.019 ^a^	0.38 ^e^
IBDQ	Control Intervention	164.9 ± 37.6 164.2 ± 34.3	170.6 ± 38.7 176.2 ± 28.0	0.149 0.001 ^a,c^	0.40 ^e^
IBDQ Digestive	Control Intervention	53.2 ± 12.8 54.5 ± 10.3	55.1 ± 12.3 55.7 ± 10.2	0.155 0.364	
IBDQ Systemic	Control Intervention	22.7 ± 6.6 22.8 ± 5.9	23.1 ± 6.8 24.3 ± 6.0	0.595 0.053	
IBDQ Social	Control Intervention	28.9 ± 6.7 27.1 ± 7.9	29.6 ± 6.5 29.9 ± 5.9	0.357 0.000 ^a,c^	0.45 ^e^
IBDQ Emotional	Control Intervention	60.1 ± 15.1 59.7 ± 13.4	62.7 ± 16.4 66.2 ± 10.2	0.103 0.000 ^a,c^	0.50 ^e^

Data are presented as mean ± standard deviation. EAE: Disease-related Stress Scale; HAD: Hospital anxiety and depression scale; PSS: Perceived Stress Scale; SRRS: Scale of Stressful Life Events; IBDQ: quality of life in IBD; ES: effect size. Significant differences using the following tests: ^a^ Paired Student’s t test; ^b^ General Linear Model control–intervention differences; ^c^ General Linear Model pre–post intervention differences; ^d^ General Linear Model control–intervention and pre–post intervention interactions; ^e^ Cohen’s d.

**Table 3:** Effectiveness of the group psychological intervention program on disease activity.

Activity	Groups	Pre-Int DS	Post-Int DS	*p*	TE (gl)
CDAI	Control	139.7 ± 98.7	97.3 ± 72.9	0.004 ^a^	0.46 (35) ^e^
	Intervention	157.6 ± 125.5	131.0 ± 111.2	0.031 ^a^	0.33 (40) ^e^
MAYO SCORE	Control	2.2 ± 2.2	1.3 ± 2.3	0.190 ^a^	(21) ^e^
	Intervention	1.8 ± 2.1	1.47 ± 2.4	0.589 ^a^	(16) ^e^

Significant differences using the following tests: ^a^ Paired Student’s t test; ^b^ General Linear Model control–intervention differences; ^c^ General Linear Model pre–post intervention differences; ^d^ General Linear Model control–intervention and pre–post intervention interactions; ^e^ Cohen’s d. TE: effect size; *p* < 0.025 applying Bonferroni correction for multiple comparisons.

**Table 4:** Effectiveness of the group psychological intervention program in activity measured by mean number of flares, number of patients with flares post-treatment, mean number of flares per month and number of flares in intervention and control arms.

	Intervention *n* = 60	Control *n* = 60	*p*	TE
Disease relapses per patient (SD)	0.2 (0.6)	0.7 (1.8)	0.027 ^a^	0.26 ^d^
Patients with disease relapses, *n* (%)	6 (10.0%)	18 (30.0%)	0.036 ^b^	0.19 ^c^
Disease relapses per month (SD)	0.03 (0.01)	0.07 (0.03)	0.034 ^e^	
Number of patients with: 1 relapse 2 relapses 3 relapses >3 relapses	4 1 0 1	8 3 3 4		

^a^ Student’s t test; ^b^ chi-squared; 1test; ^c^ Cramer v; ^d^ Cohen’s d; ^e^ Mann–Whitney U; TE: effect size.

## Data Availability

The data presented in this study are available on request from the corresponding author. The data are not publicly available due there are unpublished results.
